# Medical History from the Earliest Times

**Published:** 1893-02-04

**Authors:** E. T. Withington


### MEDICAL HISTORY FROM THE EARLIEST TIMES.

By E. T. WITHINGTON, M.A., M.B. Oxon.

### XLI.—MEDIÆVAL MILITARY MEDICINE.

SURGERY, and indeed the whole art of healing, owes much to war, that greatest of vivisectors, and this alone would justify our turning aside from time to time to consider military medicine. Tacitus tells us that the ancient Germans betook themselves after a fight to their mothers and wives, who did not hesitate to count and examine their wounds. They, doubtless, also employed some mode of treatment, though the tempting emendation of exsugere—to suck out—for exigere is not accepted by scholars. In the Lay of Nibelungs more regular practitioners make their appearance, for we hear that "those skilled in leechcraft were offered silver without weight and bright gold for healing the heroes after the battle," but this was not till they had returned home from the expedition. Some idea of the actual practice on the field may, perhaps, be gathered from an interesting passage in the companion poem, Gudrun, the German Odyssey. Here the old warrior, Wate, officiates as army surgeon, "for he had learned leechcraft from a wild woman," so he first dresses his own wounds, and then, taking a herb of marvellous power, and a plaster which he carried about with him in a box, he binds up those of his comrades; and so many did he cure, says the poet, that, had he been in a large army, "it would have taken camels to carry the gifts he would have got."

The military surgery of the Northern nations is closely similar. When St. Olaf was slain in Sticklestad fight, August, 1030, Thormod the skald was struck by an arrow in the left side, and retired to a barn, where he found women tending the wounded. One of them was boiling leeks in a pot and giving the broth to her patients, to see whether the wounds had reached the belly, for then they would smell of leeks. She asked Thormod why he did not get someone to look to his hurt, but he answered that he was not handsome enough for women to care for him, and had no presents to give. Then she took a pair of tongs and had a pull at the arrow, but failed to extract it. "Cut open the wound," said Thormod, "till you can get a good hold, and then let me pull." So he got out the iron, with bits of flesh—red and white—sticking to the barbs. "The King has fed us well, I am fat to the heart roots," observed the hero, and with that he died. Thirteen years later, King Magnus the Good of Norway and Denmark, defeated a great army of Wends near Slesnig, in one of the bloodiest battles ever fought in the North. He was concerned to find that there were not enough surgeons to look after the wounded, so he appointed those of his soldiers who had the softest hands to this office, and though they knew nothing of the healing art, they all became perfect leeches, doubtless by the aid of the King's father and patron, St. Olaf, who had wrought many healing miracles, both before and after his death. Their skill was inherited by their descendants, one of whom, Rafn Sveinbjornson, successfully performed the operation of lithotomy in the island of Iceland about 1180 A.D., in the presence of a large company, each of whom had previously repeated five Pater-nosters. But he had travelled far in his youth, even to Italy, and probably owed his skill less to St. Olaf than to Salerno. His grandson, Rafn Oddson, again appears as an army surgeon, for he went with King Erik, the Priest-hater, of Norway, on his expedition to Denmark (1289), and while tending the wounded was struck by three arrows, in the back, the arm, and the finger, the last of which caused his death. Thus, in the earlier Middle Ages, we find the wounded soldier looked after first by women, or by those of his comrades who possessed some surgical skill, and afterwards by professed surgeons, present either as forming part of the general levy, or attached to the King's person, or who had joined the army for the sake of the practice and presents they could pick up.

It is satisfactory to find that the English were not only the first of Western nations to use cannon in war, but also the first to originate some thing like an army medical service. When Prince Edward was stabbed in Palestine, it is very doubtful whether the wound was sucked by his wife; but there is good evidence that it was excised by an English surgeon, and the success of the treatment perhaps inspired him with respect for the healing art, for we find him accompanied in the invasion of Scotland (1299-1301) by no less than seven medical men. They included a king's physician and two juniors (valetti), a king's surgeon and two assistants (socii), and a simple surgeon. The king's physician and surgeon each received a knight's pay—2s. daily; and the others, who ranked as esquires, half that sum. That they found plenty to do is indicated by the fact that the chief surgeon got compensation for three horses killed in Scotland "on the King's service." But this germ of a medical staff seems to have undergone no further development, for we hear nothing of military surgeons during the wars of Edward III., except that the Welshmen who fought at Crecy were accompanied by one of their own race. In the following century appear the often-quoted names of Nicholas Colnet, physician, and Thomas Morstede, surgeon, who went with Henry V. to Agincourt. Both were attended by three mounted archers, and Morstede had, in addition, twelve members of his own craft as his assistants. Colnet and Morstede were to receive 1s., and their attendants 6d. per diem, together with a share of the plunder, and their part of "the usual bounty," viz., 100 marks (£66 13s. 4d.) per quarter for every thirty men during the actual campaign. If they got all this they were well paid indeed, but only one receipt has come down to us, in which Colnet acknowledges the payment of £8 6s. 8d. as half-quarter's salary for himself and his archers. Another surgeon, William Bredewardyn, seems to been afterwards associated with Morstede, and they were allowed two waggons and a chariot for their baggage.

Field hospitals and ambulances on a large scale appear only at the very close of the mediæval epoch, and the credit of introducing them seems to belong to that noblest of queens, Isabella the Catholic. Speaking of the siege of Alora (1484), the Spanish historian, Hernando del Pulgar, writes: "For the care of the sick and wounded the queen sent always to the camp six large tents and their furniture, together with physicians, surgeons, medicines and attendants, and commanded that they should charge nothing, for she would pay for all. These tents were called the Queen's Hospital." On the surrender of Malaga, 1487, the Spanish Army, on its entry was followed by the Queen's Hospital in 400 waggons, "ambulancias." At the siege of Granada, two years later, an eye witness, Peter Martyr, wrote to the Archbishop of Milan as follows: "Four huge hospital tents, the careful provision of queenly piety, are a sight worth seeing. They are intended not only for the wounded, but for those labouring under any disease. The physicians, apothecaries, surgeons, and other attendants are as numerous, the order, diligence, and supply of all things needful as complete as in your suburban Infirmary of the Holy Spirit or the great Milan Hospital itself. Every sickness and casualty is met and provided for by the royal bounty, except where nature's appointed day is at hand." (Regia impensa quidquid languoris, quidquid accidentis emergit, ni status cuique a natura dies adsit abscinditur.) The queen herself frequently visited the wounded, and when her courtiers hinted that this was contrary to Castilian etiquette, she is said to have replied, "Let me go to them, for they have no mothers here, and it will soothe them in their pain and weakness to find that they are not uncared for." Well may the admiring chronicler add, "Surely this queen deserved as much as those ancient Greek and Roman princesses that famous title, 'Mater castrorum.'"

